# Supplementary material for: The R2R3-MYB Transcriptional Repressor TgMYB4 Negatively Regulates Anthocyanin Biosynthesis in Tulips (Tulipa gesneriana L.)
Source: Int J Mol Sci. 2024 Jan 1;25(1):563. doi: 10.3390/ijms25010563 (PMC10779166; doi:10.3390/ijms25010563)
Supplement: Supplementary file 1 [file ijms-25-00563-s001.zip › ijms-2750248-supplementary.pdf]

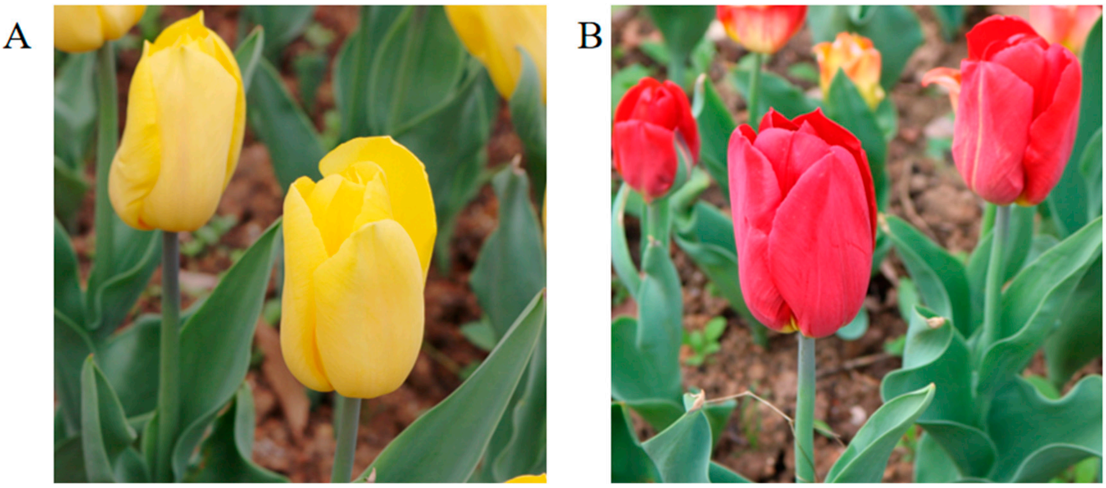

Figure S1. The henotypes of yellow petal cultivars ‘Strong Golden’ (**A**) and its budding cultivars ‘Strong Fire’ (**B**).

Table S1. The sequence of primers used in this study.

Primer	Sequence 5'-3'	Experiment
TgUBQ10-F	GTCGCACGTTGGCTGATTAC	RT-qPCR
TgUBQ10-R	GGGCTTAAAGACCACCACGA	
TgMYB4-F	GGAGCGAGGAGAAGACGATG	
TgMYB4-R	GTCCAACACTCCACTGCCAT	
NtEF1α-F	TGGTTGTGACTTTTGGTCCCA	
NtEF1α-R	ACAAACCCACGCTTGAGATCC	
NtCHS-F	AAGCAAGAGAAACTAAAGGCTACAAG	
NtCHS-R	AAATCCAAAAAGCACACCCCAT	
NtCHI-F	CGGGTGCCTCCATTCTTTTTACT	
NtCHI-R	CCTGACACTCTTTCGGCGATACTAC	
NtF3H-F	CCAGACAAACCAGATGGATGGATAG	
NtF3H-R	CAAGGGTAAGGTCGGGCTGTG	
NtF3'H-F	TGGCTATTTCATTCCAAAAGGCTCA	
NtF3'H-R	CTTCAAAGTCATTTCCTCGCACATC	
NtFLS-F	CTTGAAGGGAAAAGGGGTTGG	
NtFLS-R	CGCAACTTCTCGCAGCCTCT	
NtDFR-F	GCAGTTGCTTCCCTTTTCTACC	
NtDFR-R	TTCCCCATTGGTTGACTTTCC	
NtANS-F	GTGCCTGGGTTACAACTTTTCTATG	
NtANS-R	CATTGCTTAGGATTTCAAGGGTGTC	
TgMYB4-F	ATGGGAAGGTCTCCATGCTG	Gene cloning
TgMYB4-R	TTCCATTTCAACTGTTCTATAGTCC	
p101YFP-TgMYB4-F	CATATGGGATCTACTAGTGAATTCATGGGAAGGTCTCC ATGCTGTGAGA	Subcellular localization
p101YFP-TgMYB4-R	CCCGGGGGTACCGTCGACGGATCCCTTTTCCATTTCAA CTGTTCTATAGT	
pCAMBIA2300s-TgMYB4-F	AGCTTTCGCGAGCTCGGTACCATGGGAAGGTCTCCATG CTGTGAGA	Plant transformation
pCAMBIA2300s-TgMYB4-R	TGCCTGCAGGTCGACTCTAGACTTTTCCATTTCAACTG TTCTATAGT	
pGreenII 0800-AtDFR-F	TATAGGGCGAATTGGGTACCTTAAGCTTTTCCAAGATTT ATA	LUC
pGreenII 0800-AtDFR-R	GAACTAGTGGATCCCCCGGGTTTTGTGGTTATATGATAG ATT	
pGreenII 0800-TfANS-F	TATAGGGCGAATTGGGTACCTCTCATCCGTGGAGTCAA AC	
pGreenII 0800-TfANS-R	GAACTAGTGGATCCCCCGGGTGTTGTGAGTTGAGAGG AAG-3'	
pGreenII 62-SK-TgMYB4-F	CGCTCTAGAACTAGTGGATCCATGGGAAGGTCTCCATG CTGTGAGA	

Primer	Sequence 5'-3'	Experiment
pGreenII 62-SK-TgMYB4-R	GATAAGCTTGATATCGAATTCCTTTTCCATTTCAACTGTTCTATAGT	LUC
SK-AtPAP1-F	CGCTCTAGAACTAGTGGATCC ATGGAGGGTTCGTCCAAAGG	
SK-AtPAP1-R	GATAAGCTTGATATCGAATTC ATCAAATTTCACAGTCTCTC	
SK-TgBHLH42-1-F	CGCTCTAGAACTAGTGGATCC ATGGCCGCACCGCAGAG	
SK-TgBHLH42-1-R	GATAAGCTTGATATCGAATTC GCATAGAGAGTACTGGG	
pGADT7-TgMYB4-F	GCCATGGAGGCCAGTGAATTCATGGGAAGGTCTCCATGCTGT	Yeast assays
pGADT7-TgMYB4-R	ATTCATCTGCAGCTCGAGCTCTCATTCCATTTCAACTGTTCTATAGTCC	
pHIS-TfANS-F	ACTCACTATAGGGCGAATTCTCTCATCCGTGGAGTCA	
pHIS-TfANS-R	TAATGCCAGGAATTACTAGTTGTTGTGAGTTGAGAGG	
pHIS-AtDFR-F	ACTCACTATAGGGCGAATTCTTAAGCTTTTCCAAGAT	
pHIS-AtDFR-R	TAATGCCAGGAATTACTAGTTTTTGTGGTTATATGATA	
pGBKT7-TgMYB4-F	ATGGCCATGGAGGCCGAATTCATGGGAAGGTCTCCATGCTGT	
pGBKT7-TgMYB4-R	CTAGTTATGCGGCCGCTGCAGTCATTCCATTTCAACTGTTCTATAGTCC	
AD-TgBHLH42-1F	GCCATGGAGGCCAGTGAATTCATGGCCGCACCGCAGAGCAG	
AD-TgBHLH42-1R	CAGCTCGAGCTCGATGGATCCTTAGCATAGAGAGTACTGGG	
nLUC-bHLH2F	ACGGGGGACGAGCTCGGTACCATGGCCGCACCGCAGAG	BiLC
nLUC-bHLH2R	CGCGTACGAGATCTGGTCGAC GCATAGAGAGTACTGG	
cLuc-TgMYB4-F	TACGCGTCCCGGGGCGGTACCATGGGAAGGTCTCCATGCTGTGAGA	
cLuc-TgMYB4-R	ACGAAAGCTCTGCAGGTCGACCTTTTCCATTTCAACTGTTCTATAGT	
TRV1-F	ATTGAGGCGAAGTACGATGG	VIGS
TRV1-R	CCATCCACAATTATTTTCCGC	
TRV2-F	ATTCACTGGGAGATGATACGCT	
TRV2-R	AGTCGGCCAAACGCCGATCTCA	
TRV2-TgMYB4-F	AGTAAGGTTACCGAATTCTCTAGAAAGAACTACTGGAACACTCA	
TRV2-TgMYB4-R	CCGGGCCTCGAGACGCGTGAGCTCCTTTTCCATTTCAACTGTTCTAT	
